# Factors Influencing Nurses’ Intention and Behavior in Shared Decision-Making: A Cross-Sectional Study

**DOI:** 10.1097/jnr.0000000000000760

**Published:** 2026-08-13

**Authors:** Jeng-Cheng WU, Chih-Chi CHAN, Shih-Yuan WU, Gong-Hong LIN, Yeu-Hui CHUANG, Wen-Hsuan HOU

**Affiliations:** 1 Department of Medical Education, Taipei Medical University Hospital, Taipei, Taiwan; 2 Department of Urology, Taipei Medical University Hospital, Taipei, Taiwan; 3 Department of Education and Humanities in Medicine, School of Medicine, College of Medicine, Taipei Medical University, Taipei, Taiwan; 4 Department of Urology, School of Medicine, College of Medicine, Taipei Medical University, Taipei, Taiwan; 5 TMU Research Center of Urology and Kidney, Taipei Medical University, Taipei, Taiwan; 6 Department of Health Promotion and Health Education, College of Education, National Taiwan Normal University, Taipei, Taiwan; 7 School of Medicine, College of Medicine, Taipei Medical University, Taipei, Taiwan; 8 Master Program in Long-Term Care, College of Nursing, Taipei Medical University, Taipei, Taiwan; 9 International Ph.D. Program in Gerontology and Long-Term Care, College of Nursing, Taipei Medical University, Taipei, Taiwan; 10 School of Nursing, College of Nursing, Taipei Medical University, Taipei, Taiwan; 11 Research Center in Nursing Clinical Practice, Wan Fang Hospital, Taipei Medical University, Taipei, Taiwan; 12 Department of Physical Medicine and Rehabilitation, Taipei Medical University Hospital, Taipei, Taiwan; 13 Department of Physical Medicine and Rehabilitation, School of Medicine, College of Medicine, Taipei Medical University, Taipei, Taiwan; 14 Graduate Institute of Clinical Medicine, College of Medicine, Taipei Medical University, Taipei, Taiwan; 15 School of Gerontology and Long-Term Care, College of Nursing, Taipei Medical University, Taipei, Taiwan; 16 Cochrane Taiwan, Taipei Medical University, Taipei, Taiwan; † Contributed equally

**Keywords:** shared decision-making, nurse, theory of planned behavior, intention, behavior

## Abstract

**Background:**

Shared decision-making (SDM), a cornerstone of patient-centered care, is associated with increased patient satisfaction and improved health outcomes. Nurses, as integral members of the health care team, play a pivotal role in SDM by advocating for patients and facilitating communication. However, their involvement in SDM is often constrained by systemic and organizational factors. Identifying the factors that significantly influence nurses’ engagement in SDM is essential to advancing SDM practice.

**Purpose:**

This study, guided by the theory of planned behavior (TPB), was designed to explore the factors of influence on the intentions and perceived behaviors of nurses related to SDM.

**Methods:**

A cross-sectional study was conducted on 159 nurses recruited through convenience sampling from 11 teaching hospitals in Taiwan. The data used in the analyses were collected using a self-administered questionnaire developed to measure the following TPB constructs: intention, attitude, subjective norms, perceived behavioral control, and perceived SDM behavior. Descriptive statistics, univariate analyses, and hierarchical linear regression were performed to examine the associations among TPB variables, intention, and perceived SDM behavior.

**Results:**

A significant correlation was identified between age and intention to engage in SDM (Pearson’s *r* = .250, *p* = .001). Participants with a master’s degree or higher qualification reported significantly stronger intention to participate in SDM than those holding either an associate or bachelor’s degree (3.58 vs. 1.29, *p* < .001). The mean perceived SDM behavior score was 12.86 (*SD* = 4.25). A hierarchical regression analysis found attitude (*β* = 0.40, *p* < .001) and perceived behavioral control (*β* = 0.35, *p* < .001) to significantly predict intention to engage in SDM (adjusted *R* ^2^ = .436, *p* < .001). In addition, all of the TPB variables were identified as significant predictors of perceived SDM behavior. The final regression model explained 48.8% of the variance (*p* < .001). Exhibiting a more positive attitude, greater perceived control, and stronger intention were each associated with having a higher level of perceived SDM behavior.

**Conclusions/Implications for Practice:**

The findings of this study underscore the importance of intention and behavior in the participation in SDM by nurses, with attitude and perceived behavioral control identified as key determining factors. Thus, these factors, together with intention to engage in SDM, significantly influence perceived SDM behavior. Hospital managers and educators should implement comprehensive strategies, including related initiatives, skill training, organizational support, and incentives, to increase participation by nurses in SDM.

## Introduction

Shared decision-making (SDM) is a collaborative approach in which clinicians and the patient make health care decisions together, allowing the integration of the best available evidence with the patient’s values and preferences (Elwyn, 2021). This approach marks a shift from the traditional paternalistic model, in which clinicians primarily direct decision-making, to a partnership model, in which patients are empowered as active decision-makers (Tariman et al., 2018). SDM has gained recognition as the preferred framework for patient-centered care, with research linking it to higher decision quality, greater patient satisfaction, and better care experiences (Stiggelbout et al., 2015).

In clinical practice, nurses have traditionally served as communicators and educators rather than active participants in patient decision-making processes. Nevertheless, nurses play a pivotal role in the SDM process as front-line communicators who translate complex medical information into understandable language, advocate for patient concerns, and offer continual support (Tariman et al., 2018). In addition, their extended interaction with patients and their families leads to close relationships and positions them as trusted health care providers. This trust enables nurses to elicit patients’ values and preferences and advocate for these preferences within the interdisciplinary team (Faiman & Tariman, 2019). Millard et al. (2006) highlighted the nurse–patient relationship as the key channel for SDM, noting that effective communication and trust facilitate patient involvement in decisions regarding the timing of home visits and care related to activities of daily living. Through SDM, nurses not only align care with patient preferences but also gain greater control over their practice, improve their job satisfaction, and contribute to better care outcomes at the organizational level (Chung et al., 2021).

Despite the critical role that nurses play in SDM, their involvement in real-world SDM processes remains limited. SDM-related responsibilities are still often placed primarily on physicians, with the contributions to SDM of nurses frequently undervalued (Olling et al., 2021). This limited engagement stems from not only individual factors but also broader social and organizational challenges. Systemic challenges commonly cited include time constraints and heavy workloads, which restrict opportunities for the in-depth communication required for SDM to succeed (Chung et al., 2021; Chung et al., 2019). Role ambiguity and hierarchical team structures further inhibit engagement, as nurses may perceive treatment decision-making as being outside their professional scope or fear overstepping traditional boundaries in the physician-led decision-making process. Furthermore, lack of formal SDM training and limited exposure to tools such as decision aids hinder the readiness of nurses to participate effectively (Chung et al., 2019; Friedberg et al., 2013). Moreover, workplace cultural factors and an unsupportive organizational environment may further decrease the willingness of nurses to collaborate with patients through SDM. These barriers contribute to inconsistent and often inadequate nurse participation in SDM.

Although these barriers are well documented, the focus of most related studies has been limited to identifying obstacles or qualitatively describing nurse attitudes and perceptions toward SDM. Few scholars have examined systematically the determinants of SDM behaviors in nurses using a validated theoretical framework. In particular, quantitative research into how interactions among individual, social, and organizational factors affect the intention and actual behavior of nurses with regard to SDM is lacking in the literature. Truglio-Londrigan (2013) noted the limited research on SDM within the nursing field, reporting that SDM has only rarely been examined comprehensively from the perspective of nurses.

The theory of planned behavior (TPB) provides an evidence-based model for understanding SDM dynamics because it delineates how attitude, subjective norms, and perceived behavioral control shape behavioral intention and actual action (Ajzen, 1991; Armitage & Conner, 2001). According to the TPB, an individual’s intention to perform a behavior is shaped by three key factors: their attitude (positive or negative evaluation of the behavior), subjective norms (perceived social pressure or support for engagement in the behavior), and perceived behavioral control (perceived ease or difficulty of performing the behavior, including confidence and access to resources). Both behavioral intention and perceived behavioral control have been identified in the literature as predictors of actual behavior. In other studies, applications of the TPB to SDM have predominantly centered on physicians, with limited attention given to nurses, despite their essential role in patient care (Thompson-Leduc et al., 2015). To address this gap, in this study, the TPB framework was employed to examine the factors influencing intention and behaviors related to SDM among nurses in Taiwan. The findings provide preliminary insights into how attitude, subjective norms, and perceived behavioral control relate to perceived SDM behaviors in a health care context characterized by hierarchical medical structures and evolving SDM policies that present unique challenges and opportunities. The findings of this study are intended to provide a reference for future efforts to support the greater engagement of nurses in SDM.

## Methods

### Research Design, Setting, and Participants

A cross-sectional study was conducted in 2023 across 11 teaching hospitals currently participating in SDM campaigns. Convenience sampling was used to recruit nurses with experience in implementing SDM as participants. Currently working nurses who were 20 years or older and had a professional nursing license and more than one year of clinical experience at their current institution were eligible to participate. The sample size was determined using the approach described in Keshmiri et al. (2020), who applied a TPB framework to investigate the impact of intention to engage in SDM in emergency care on health care professionals, with a reported effect size of 0.14. To achieve a type I error rate (α) of .05 and a statistical power of 0.8, and assuming an estimated nonresponse rate of 10%, a minimum sample of 116 participants was required for this study.

### Measures

The self-administered questionnaire used in this study was developed by Guerrier et al. (2013) using the theoretical constructs of the TPB, with questionnaire items targeting demographic variables (e.g., sex, age, educational level, and administrative role) and TPB-related factors, including intention to engage in SDM, attitude toward SDM, subjective norms, perceived behavioral control, and perceived SDM behavior. The SDM questionnaire used in this study was authored by France Légaré (2013) and originally designed to assess behavioral intention in physicians and related determinants. This questionnaire comprises four TPB constructs, including intention to engage in SDM, attitude toward SDM, subjective norms, and perceived behavioral control. The 11 TPB-related items on the questionnaire are rated on a 7-point Likert Scale ranging from −3 to +3, with higher scores indicating more favorable intention, attitude, subjective norms, and perceived behavioral control in SDM.

### Intention to Engage in Shared Decision-Making

Intention, referring to an individual’s perceived likelihood of performing a specific behavior, reflects their motivation as well as active planning/decision-making with regard to executing related actions (Conner & Armitage, 1998). To assess the motivation and willingness of the participants to engage in SDM during patient consultations, two items measuring intention were administered to separately assess the degree of motivation and commitment to performing SDM-related behaviors.

### Attitude Toward Shared Decision-Making

Attitude refers to the degree to which an individual perceives a behavior as favorable or unfavorable, as well as to the positive or negative feelings associated with performing that behavior, which is used to proxy the individual’s evaluation (i.e., degree of favorability) and the anticipated outcomes of engaging in that behavior, with people preferring actions that lead to desirable results (Ajzen, 1991). Attitudes are usually expressed in emotions, beliefs, and personal behaviors. In this study, attitude toward SDM was assessed using four items that captured professional judgments regarding and emotional responses to SDM, including perceived responsibility, desirability, sense of fulfillment or satisfaction, and potential frustration related to engaging in SDM.

### Subjective Norms

Subjective norms, which refer to the perceived social pressure to perform or refrain from specific behaviors, are shaped by approval, support, or disapproval from significant others (e.g., friends, family, colleagues, supervisors) and influence the performance of these behaviors (Ajzen, 1991, 2020; Keshmiri et al., 2020; Légaré et al., 2013). Three of the questionnaire items were designed to assess the perceived influence of significant others in terms of their recommending, encouraging, or preferring the participant to engage in SDM.

### Perceived Behavior Control

Perceived behavioral control refers to an individual’s assessment of their ability to manage future situations, with related behaviors being strongly influenced by confidence in their ability to perform the behavior (Ajzen, 1991). One of the questionnaire items was designed to assess perceived situational barriers that may hinder engagement in SDM, while one other was designed to evaluate self-efficacy and confidence in the participant’s ability to successfully implement SDM in clinical practice.

### Perceived Shared Decision-Making Behavior

The five-item Observer OPTION^5^ scale (Dillon et al., 2017) used in this study to assess self-reported confidence in participant actions during the SDM process is an abbreviated version of the original 12-item instrument. Scoring is done on a 5-point Likert Scale ranging from 0 to 4. Perceived SDM behavior is evaluated across the four key facets of action, target, context, and time. This scale has been employed in previous research to assess health care professionals’ delivery of each step of the SDM process, with good internal consistency (Cronbach α = .92) and criterion validity (Person's *r* = .69, *p* = .01) demonstrated (Hsiao et al., 2022). The task descriptions are provided in Table 1.

**Table 1:** Item-Level Analysis of Perceived Shared Decision-Making Behavior Measured Using the OPTION^5^ Scale (*N* = 159)

Item	Task Description	Mean	*SD*	Order
1. Presenting options	For the health issue being discussed, I draw attention to or confirm that alternate treatment or management options exist or that the need for a decision exists (if the patient rather than I draws attention to the availability of options, I respond by agreeing that the options need deliberation)	2.58	0.96	2
2. Patient partnership	I reassure the patient or reaffirm that I will support their becoming more informed or deliberate about the options (if the patient states they have sought or obtained information prior to the encounter, I support such a deliberation process)	2.48	1.02	5
3. Describing pros/cons	I give information or check understanding about the options that are considered reasonable (this can include taking no action) to support the patient in comparing alternatives (if the patient requests clarification, I support the process)	2.70	0.96	1
4. Eliciting preferences	I make an effort to elicit the patient's preferences in response to the options that have been described (if the patient declares their preference(s), I am supportive)	2.58	1.05	3
5. Integrating patient preferences	I make an effort to integrate the patient’s elicited preferences as decisions are made (if the patient indicates how best to integrate their preferences as decisions are made, I make an effort to do so)	2.52	0.97	4

*Note.* OPTION^5^ = Observing Patient Involvement in Decision-Making–5-item version.

### Validity and Reliability

Favorable reliability and validity were previously demonstrated for all of the aforementioned scales (Dillon et al., 2017; Guerrier et al., 2013). In this study, the Cronbach’s α values for the intention, attitude, subjective norm, and perceived behavioral control scales were .947, .870, .959, and .848, respectively. The Mandarin version of the Observer OPTION^5^ scale was demonstrated to have satisfactory reliability, with a Cronbach’s α value of .910. Both scales (the TPB scale and the OPTION^5^ scale) were translated from English into Mandarin Chinese using a back-translation approach.

### Data Collection Procedure

For data collection, a self-reported, anonymous questionnaire was administered through the online SurveyCake platform from August 2023 to November 2023. Nurses with at least one year of clinical experience were recruited through the quality management centers of 11 teaching hospitals located in northern, central, southern, and eastern Taiwan. These quality management centers were contacted using a standard request for assistance in disseminating the survey link and QR code through their internal platforms (e.g., LINE and email) along with an invitation that stated the study’s purpose, voluntary nature, estimated completion time, and assurance of anonymity. Informed consent was obtained electronically from all participants. The study protocols were reviewed and approved by the Human Research Ethics Committee of Taipei Medical University Joint Institutional Review Board (TMU-JIRB; N202212064).

### Statistics

All statistical analyses were conducted using IBM SPSS Statistics 24.0 (IBM Corp., Armonk, NY, USA). Categorical data were presented as frequencies and percentages, and continuous data were summarized using means, SDs, and minimum and maximum values. Univariate analyses, including independent sample *t* tests and Pearson correlation analysis, were conducted to examine associations between demographic and outcome variables, with a *p*-value of <.05 considered statistically significant. Hierarchical linear regression analyses were then performed to examine the relationships between TPB-related factors and perceived SDM behavior among the participants.

## Results

One hundred and fifty-nine nurses (mean age: 38.46 years) participated in this study. As shown in Table 2, nearly all of the participants were females (96.2%); most (79.2%) held either an associate or bachelor’s degree, while 20.8% held a master’s degree or higher; and 26.4% worked in administrative positions. The range, mean, and *SD* of scores for all 11 items covering the TPB attitude, subjective norms, perceived behavioral control, and intention to engage in SDM constructs are also given in Table 3.

**Table 2:** Baseline Participant Characteristics (*N* = 159)

Variable	*n*	%
Age (y); *M* (*SD*)	38.46	7.90
Range	24 to 58	
Sex		
Female	153	96.2
Male	6	3.8
Educational level		
Associate or bachelor’s degree	126	79.2
Master’s degree or higher	33	20.8
Administrative role		
Yes	42	26.4
No	117	73.6
Affiliated hospital level		
Medical center	41	25.8
Regional hospital	90	56.6
District hospital	28	17.6
Nursing specialty		
Internal medicine	80	50.3
Surgery	48	30.2
Obstetrics and gynecology	3	1.9
Pediatrics	6	3.8
Emergency medicine	8	5.0
Others	14	8.8

**Table 3:** Scores for Participants’ Perceived SDM Behavior, Intention to Engage in SDM, and TPB-Related Factors

Variable	Theoretical Range	Data Range	Mean	*SD*
**TPB-related factors**				
Attitude toward SDM (total score)	−12 to 12	−2 to 12	6.22	3.45
Attitude toward SDM (average score)	−3 to 3	−0.5 to 3	1.56	0.86
1. When a decision needs to be made during a consultation. I think engaging in shared decision-making would be a responsible thing to do	−3 to 3	0 to 3	1.78	1.03
2. When a decision needs to be made during a consultation. I think engaging in shared decision making would be pleasant	−3 to 3	0 to 3	1.35	0.98
3. When a decision needs to be made during a consultation, I think engaging in shared decision making would be worthwhile	−3 to 3	−1 to 3	1.76	1.03
4. When a decision needs to be made during a consultation, I think engaging in shared decision making would be gratifying	−3 to 3	−2 to 3	1.33	1.02
Subjective norms (total score)	−9 to 9	−9 to 9	3.38	3.97
Subjective norms (average score)	−3 to 3	−3 to 3	1.13	1.32
5. Most of the people who are important to me would recommend that I engage in shared decision making when a decision needs to be made during a consultation	−3 to 3	−3 to 3	1.14	1.36
6. Most of the people who are important to me are favorable that I engage in shared decision making when a decision needs to be made during a consultation	−3 to 3	−3 to 3	1.13	1.43
7. Most of the people who are important to me would think it preferable that I engage in shared decision making when a decision needs to be made during a consultation	−3 to 3	−3 to 3	1.10	1.34
Perceived behavioral control (total score)	−6 to 6	−5 to 6	1.26	2.55
Perceived behavioral control (average score)	−3 to 3	−2.5 to 3	0.63	1.27
8. I feel I would be capable of engaging in shared decision making when a decision needs to be made during a consultation	−3 to 3	−3 to 3	0.83	1.31
9. I don’t see any obstacles to engaging in shared decision making when a decision needs to be made during a consultation	−3 to 3	−3 to 3	0.43	1.42
Intention to engage in SDM (total score)	−6 to 6	−6 to 6	1.77	2.81
Intention to engage in SDM (average score)	−3 to 3	−3 to 3	0.88	1.41
10. When a decision needs to be made during a consultation, what are the chances that you would engage in shared decision making	−3 to 3	−3 to 3	0.92	1.44
11. When a decision needs to be made during a consultation, I intend to engage in shared decision making	−3 to 3	−3 to 3	0.84	1.44
**Perceived SDM behavior (OPTION^5^)**	0 to 20	0 to 20	12.86	4.25

*Note.* SDM = shared decision-making; TPB = theory of planned behavior; OPTION^5^ = Observing Patient Involvement in Decision-Making—5-item version.

The mean perceived SDM behavior score, as assessed using the OPTION^5^ scale, was 12.86 (*SD* = 4.25, range = 0 to 20). The 5-item OPTION^5^ scale, summarized in Table 1, is a concise assessment tool based on the three-talk model that comprises the following five core clinical behaviors indicative of SDM: (1) constructive interpersonal engagement, (2) recognition of alternative actions, (3) comparative learning, (4) preference construction and elicitation, and (5) preference integration (Elwyn et al., 2021). Each item is scored on a 5-point Likert Scale ranging from 0 (*not observed*) to 4 (*executed to a high standard*), with higher scores indicating greater performance in SDM (Barr et al., 2015). In terms of the mean scores for the five OPTION^5^ items in this study, the highest was “giving information or checking understanding about the options that are considered reasonable” (mean = 2.70, *SD* = 0.96), while the lowest was “providing reassurance or reaffirmation about the options” (mean = 2.48, *SD* = 1.02).

The results of the univariate analysis used to examine the associations among perceived SDM behavior, intention, and TPB-related factors are presented in Table 4. Age was found to correlate significantly with attitude toward SDM (*r* = .299, *p* < .001), subjective norms (*r* =.211, *p* = .007), and intention to engage in SDM (*r* = .250, *p* = .001). Participants with a master’s degree or higher earned a significantly higher mean score than those without a graduate education for all TPB-related factors, that is, attitude toward SDM, subjective norms, perceived behavioral control, intention to engage in SDM, and perceived SDM behavior (7.61 ± 2.77 vs. 5.86 ± 3.52, 4.91 ± 3.78 vs. 2.98 ± 3.93, 2.58 ± 2.82 vs. 0.92 ± 2.37, 3.58 ± 2.00 vs. 1.29 ± 2.81, and 14.30 ± 3.85 vs. 12.48 ± 4.28, respectively). In addition, those in administrative roles earned a significantly higher mean score than their peers in other roles across the same five TPB-related factors (7.81 ± 2.53 vs. 5.65 ± 3.56, 4.79 ± 3.98 vs. 2.87± 3.86, 2.36 ± 2.66 vs. 0.87 ± 2.40, 3.17 ± 2.05 vs. 1.26 ± 2.89, and 14.26 ± 3.86 vs. 12.36 ± 4.28, respectively).

**Table 4:** Univariate Analysis of Participants’ SDM Behavior, Intention to Engage in SDM, and TPB-Related Factors

Variable	Attitude Toward SDM		Subjective Norm		Perceived Behavioral Control		Intention to Engage in SDM		Perceived SDM Behavior (OPTION^5^)	
	Mean±*SD*/Pearson *r*	*p*	Mean±*SD*/ Pearson *r*	*p*	Mean±*SD*/ Pearson *r*	*p*	Mean±*SD*/ Pearson *r*	*p*	Mean±*SD/*Pearson *r*	*p*
**Personal background factor**										
Age (y)	.299	<.001	.211	.007	.128	.107	.250	.001	.122	.126
Sex										
Female	6.18±3.47	.421	3.35±3.98	.621	1.25±2.52	.818	1.80±2.80	.409	12.77±4.27	.176
Male	7.33±2.66		4.17±3.87		1.50±3.45		0.83±3.37		15.17±2.93	
Educational level										
Associate or bachelor’s degree	5.86±3.52	.003	2.98±3.93	.012	0.92±2.37	.001	1.29±2.81	<.001	12.48±4.28	.028
Master’s degree or higher	7.61±2.77		4.91±3.78		2.58±2.82		3.58±2.00		14.30±3.85	
Administrative role										
Yes	7.81±2.53	<.001	4.79±3.98	.007	2.36±2.66	.001	3.17±2.05	<.001	14.26±3.86	.012
No	5.65±3.56		2.87±3.86		0.87±2.40		1.26±2.89		12.36±4.28	
**TPB-related factor**										
Attitude toward SDM	—		.502	<.001	.419	<.001	.560	<.001	.557	<.001
Subjective norms	—		—		.483	<.001	.358	<.001	.515	<.001
Perceived behavioral control	—		—		—		.535	<.001	.575	<.001
**Intention to engage in SDM**	—		—		—		—		.547	<.001

*Note.* SDM = shared decision-making, TPB = theory of planned behavior, OPTION^5^ = Observing Patient Involvement in Decision-Making—5-item version.

The results of the hierarchical linear regression analysis of intention to engage in SDM are summarized in Table 5. In model 1, personal background factors explained 11.6% of the variance in SDM intention (*R* ^2^ = .138, adjusted *R* ^2^ = .116, *p* < .001), with educational level the only factor to reach statistical significance (β = 0.22, *p* = .021). After adjusting for personal background factors in the regression model (model 3), attitude toward SDM (β = 0.40, *p* < .001) and perceived behavioral control (β = 0.35, *p* < .001) were identified as significant predictors of intention to practice SDM, with subjective norms failing to reach statistical significance. The final model explained 43.6% of the variance in SDM intention (*R* ^2^ = .461, adjusted *R* ^2^ = .436, *p* < .001).

**Table 5:** Hierarchical Linear Regression Analysis of TPB-Related Factors Predicting Intention to Engage in SDM

Variable	Model 1		Model 2		Model 3		Model 4		Model 5		VIF
	*β*	*p*	*β*	*p*	*β*	*p*	*β*	*p*	*β*	*p*	
**Personal background factor**											
Age (year)	0.10	.229	0.00	.958	0.07	.416	0.12	.127	0.03	.635	1.417
Sex (reference: male)	0.08	.312	0.11	.085	0.09	.228	0.08	.206	0.11	.076	1.020
Educational level (reference: associate or bachelor’s degree)	0.22	.021	0.22	.008	0.20	.030	0.14	.096	0.17	.036	1.697
Administrative role (reference: no administrative role)	0.11	.279	0.02	.830	0.08	.419	0.03	.734	0.01	.864	1.825
**TPB-related factor**											
Attitude toward SDM			0.52	<.001					0.40	<.001	1.508
Subjective norms					0.29	<.001			−0.04	.545	1.531
Perceived behavioral control							0.48	<.001	0.35	<.001	1.446
*F*	6.160		18.307		8.456		16.180		18.448		
*p*	<.001		<.001		<.001		<.001		<.001		
*R*	.371		.612		.465		.588		.679		
*R* ^*2*^	.138		.374		.217		.346		.461		
Adjusted *R* ^*2*^	.116		.354		.191		.324		.436		
*ΔR* ^*2*^			.236		.079		.208		.323		

*Note.* SDM = shared decision-making, TPB = theory of planned behavior. Model 1: Personal background factors; Model 2: Personal background factors + attitude toward SDM; Model 3: Personal background factors + subjective norms; Model 4: Perceived behavioral control; Model 5: Personal background factors + attitude toward SDM + Subjective norms + perceived behavioral control.

The results of the hierarchical linear regression analysis of perceived SDM behavior are summarized in Table 6. In model 1, personal background factors explained 3.2% of the variance in SDM behavior (*R* ^2^ = .056, adjusted *R* ^*2*^= .032, *p* = .062), but the model was not statistically significant, indicating these factors alone are insufficient predictors. In model 2, the inclusion of TPB-related factors increased the explanatory power significantly (Δ*R* ^2^ = .428, *p* < .001). Attitude toward SDM (β = 0.33, *p* < .001), subjective norms (β = 0.19, *p* = .010), and perceived behavioral control (β = 0.35, *p* < .001) were identified as significant predictors. In model 3, which included intention to engage in SDM, attitude toward SDM (β = 0.23, *p* = .003), perceived behavioral control (β = 0.27, *p* < .001), and subjective norms (β = 0.20, *p* = .005) remained significant, with intention also identified as a significant predictor of perceived SDM behavior (β = 0.24, *p* = .003). The final model accounted for 48.8% of the variance in perceived SDM behavior (adjusted *R* ^2^ = .488, *p* < .001).

**Table 6:** Hierarchical Linear Regression Analysis of TPB-Related Factors Predicting Perceived SDM Behavior

Variable	Model 1			Model 2			Model 3		
	*β*	*p*	VIF	β	*p*	VIF	β	*p*	VIF
Personal Background Factor									
Age (years)	0.02	.838	1.347	−0.07	.346	1.417	−0.07	.278	1.419
Sex (Reference: Male)	−0.11	.164	1.015	−0.08	.204	1.020	−0.10	.085	1.042
Educational level (Reference: Associate or bachelor’s degree)	0.07	.513	1.659	−0.01	.871	1.697	−0.05	.495	1.747
Administrative role (Reference: No administrative role)	0.15	.143	1.779	0.02	.812	1.825	0.02	.774	1.826
TPB-related factor									
Attitude toward SDM				0.33	<.001	1.508	0.23	.003	1.811
Subjective norms				0.19	.010	1.531	0.20	.005	1.535
Perceived behavioral control				0.35	<.001	1.446	0.27	<.001	1.667
Intention to engage in SDM							0.24	.003	1.855
*F*	2.288			20.223			19.835		
*p*	.062			<.001			<.001		
*R*	.237			.696			.717		
*R* ^*2*^	.056			.484			.514		
Adjusted *R* ^*2*^	.032			.460			.488		
*R* ^*2*^ change				.428			.030		

*Note.* SDM = shared decision-making, TPB = theory of planned behavior. Model 1: Personal background factors; Model 2: Personal background factors + TPB-related factors; Model 3: Personal background factors + TPB-related factors + intention to engage in SDM.

Univariate analyses were also conducted to assess the direct and indirect relationships between the TPB-related factors, revealing that all TPB-related factors (i.e., attitude toward SDM, subjective norms, and perceived behavioral control) both directly affected perceived SDM behavior and indirectly mediated this behavior through intention to engage in SDM. In addition, the comprehensive TPB framework includes both direct and indirect pathways between the TPB-related factors and perceived SDM behavior. The only nonsignificant path identified was the association between subjective norms and intention to engage in SDM.

## Discussion

### Main Findings

To the best of the authors’ knowledge, this was the first study to use the TPB framework to examine the factors influencing perceived SDM behavior in nurses. In this study, only attitude and perceived behavioral control were identified as significantly associated with intention to engage in SDM. However, all of the TPB-related factors (attitude, subjective norm, and perceived behavioral control) as well as intention to engage in SDM were found to be significantly associated with perceived SDM behavior. These findings are consistent with the TPB framework and may be used to guide the development of strategies for enhancing the participation of nurses in SDM.

### Descriptive Statistics

Average TPB-related factor scores ranked from highest to lowest were: attitude (1.56), subjective norms (1.13), intention to engage in SDM (0.88), and perceived behavioral control (0.63). By contrast, in a prior survey of Canadian physicians, the average scores ranked from highest to lowest were: intention (1.77), perceived behavioral control (1.6), subjective norms (1.4), and attitude (1.2). The larger mean difference in the Canadian study for intention and perceived behavioral control (0.82 and 0.97, respectively; Guerrier et al., 2013) may reflect differences in professional roles, health care system structures, and/or cultural expectations regarding SDM implementation (Bos-van den Hoek et al., 2021). Most of the nurses in this study gave relatively low scores for each domain of the TPB framework. Also, the highest negative skew presentation was in the construct of subjective norm (i.e., very close to 1), which may reflect the lower social pressure perceived by nurses to perform the SDM process due to the expectations or disapproval of friends, family, and colleagues. In the assessment of perceived SDM behavior, the participants in this study scored highest in providing alternative options and describing pros and cons (2.70) and lowest in building partnerships through the process of deliberation to inform patients (2.48). This result is similar to a Dutch study designed to assess SDM performance among medical residents using OPTION^5^ scores. In that study, the residents scored highest in describing the pros and cons of alternative options and lowest in patient partnership deliberation (Baghus et al., 2024), suggesting health care professionals generally devote minimal effort to deliberating with and supporting patients with regard to their decision-making options. Variation in perceived SDM behavior has been observed among studies that have used the same tool to evaluate various health care professionals such as general physicians, physical therapists, and dieticians (Hacquebord et al., 2025; Vaillancourt et al., 2015; Williams et al., 2019). This variation is attributable to the use of self-rated measures to assess perceived SDM behavior. In addition, the participants in this study were nurses with prior experience in SDM, which may have positively affected their self-perceptions, leading them to overestimate their SDM self-efficacy. Moreover, Observer OPTION^5^ assessments have exhibited substantial variation across clinical fields (Dillon et al., 2017).

### Factors Influencing Shared Decision-Making Intention

In this study, attitude toward SDM had the strongest impact on intention to engage in SDM, highlighting the critical role of personal beliefs and perceptions in shaping motivation. This finding is consistent with the TPB framework, which highlights attitude as a primary determinant of behavioral intention (Ajzen, 1991; Armitage & Conner, 2001). Similar findings have been reported in other studies of nursing behavior. For example, Dionisi et al. (2020) demonstrated that the attitude of nursing students toward medication safety significantly influenced the likelihood of their reporting errors, with positive attitudes enhancing both reporting rate and professional accountability. Similarly, Tsemach and Aharon (2025) demonstrated attitude to be a significant predictor of nurse involvement in euthanasia decision-making, with those holding more favorable views being more willing, despite systemic and ethical barriers, to engage in SDM. The results of this study are consistent with those of another study that found nurses holding more positive attitudes toward SDM to be more likely to incorporate SDM into clinical practice (Hsu et al., 2021). In addition, a qualitative study of clinicians in a hospital that successfully implemented SDM reported that strong leadership helped overcome initial skepticism and reinforced positive SDM behaviors (Ankolekar et al., 2021). The above findings underscore the pivotal role of attitude in shaping the perspectives of nurses regarding the importance of engaging in SDM. Although subjective norms were found to correlate significantly with SDM intention in the univariate analysis, the correlation failed to reach significance in the final regression model. This finding suggests that, although social and organizational expectations shape inclination toward SDM in nurses, their ultimate intention to engage in SDM is affected primarily by personal attitude and perceived behavioral control rather than external social influences from patients or peers. This pattern is consistent with findings from another study, which emphasized that, although subjective norms significantly influence health care behavioral intention, the effect size is relatively small compared with other factors (Maleki et al., 2025). However, the results in this study contrast with those from a systematic review focused mainly on physicians that identified subjective norms as a key determinant of SDM intention, with a significant association found in 15 of the 21 analyses (Thompson-Leduc et al., 2015). This discrepancy may be attributable to the differing roles of physicians and nurses in clinical decision-making. Physicians are typically the primary decision-makers, while nurses often play a supportive or complementary role in SDM, especially in complex cases, due to their limited time and high workload (Arends et al., 2023).

### Factors Influencing Perceived Shared Decision-Making Behavior

The results of this study suggest that nurses in Taiwan generally engage in only some components of the SDM process. Although most of the participants were able to provide information on available and alternative treatment options, relatively few demonstrated behaviors such as active listening, thorough deliberation, and the integration of patient preferences into final decisions. Williams et al. (2019) reported that clinicians in the United Kingdom also struggle with the more interactive aspects of SDM, which received the lowest scores. Similar findings obtained using the OPTION^5^ scale were also reported in a Dutch study, with average SDM performance reported as 34 out of 100 and the lowest and highest scores earned for, respectively, supporting patient deliberation and providing information about treatment options (Peters et al., 2022). One possible explanation for these findings in this study is grounded in the specific dynamics of the Taiwanese health care system, in which physicians are considered the primary decision-makers and nurses regularly assume roles as communicators and educators, and do not actively participate in decision-making. Even with the recent promotion of SDM initiatives, cultural and professional hierarchies remain influential. In Taiwan, nurses typically operate within a structured hierarchy in which physicians lead medical decisions, and nurses are expected to support these decisions. This hierarchical structure may limit the extent to which Taiwanese nurses engage in key SDM behaviors (Chung et al., 2021).

The findings of this study indicate that intention to engage in SDM influences perceived SDM behavior significantly. This is consistent with the TPB framework, which posits that individuals with stronger behavioral intentions are more likely to translate this intention into action (Ajzen, 1991; Armitage & Conner, 2001). This underscores the importance of intention in facilitating the adoption of SDM practices among nurses. For example, Ko et al. (2004) demonstrated that nurses’ intention to care for patients with severe acute respiratory syndrome significantly influenced their actual engagement in patient care, emphasizing the role of motivation in translating intent into behavior. These findings suggest that future efforts to improve SDM implementation should prioritize strengthening structured training programs, implementing supportive institutional policies, and the provision of decision aids to increase nurse knowledge and promote positive attitudes, skills, and self-efficacy (Bos-van den Hoek et al., 2023; Poulin Herron et al., 2022).

The results of this study further indicate that perceived behavioral control influences not only intention to engage in SDM but also self-perceived SDM behavior, as this control determines the confidence nurses have in performing SDM as well as their self-perceived ability to overcome barriers. This suggests that, beyond the formation of intention, confidence in one’s ability to perform SDM and the availability of necessary infrastructural resources are essential for SDM implementation in nurses. These results are consistent with previous TPB-based studies conducted in health care settings. For example, J. Wang et al. (2021) reported perceived behavioral control directly predicted the use by nurses of physical restraints in long-term care facilities, highlighting the role of perceived behavioral control in determining actual behavior beyond intention. Similarly, Piras et al. (2018) demonstrated that adherence by nurses to hand hygiene protocols is significantly influenced by perceived behavioral control, reinforcing the notion that self-efficacy and external resources play crucial roles in clinical decision-making. Taken together, these findings underscore the importance of addressing both individual and systemic determinants to promote SDM engagement among nurses. Future interventions should focus on enhancing nurses’ perceived behavioral control through targeted training, supportive institutional policies, and the provision of adequate resources to not only strengthen intention to practice SDM but also improve the likelihood of its consistent execution in clinical practice.

Although subjective norms were not shown to significantly predict intention to engage in SDM, they directly affected perceived SDM behavior in the hierarchical linear regression model. While this finding does not conform to the original TPB framework, several variations of this framework have been validated for describing the clinical behavior of health care personnel (Keshmiri & Salar, 2018; Vaillancourt et al., 2015; D. Wang et al., 2021; J. Wang et al., 2021). The results of other analyses conducted in this study revealed subjective norms to be associated with perceived SDM behavior both directly and through intention to engage in SDM as a mediator. This finding suggests that, compared with other health care professionals, nurses may be less likely to engage in SDM due to inadequate peer support as well as social norms/expectations (Allen et al., 2020). Thus, nurses may be motivated to engage in SDM to conform to workplace norms or avoid social disapproval, thereby bypassing the internal process of intention formation.

### Limitations

This study was affected by several limitations. First, study data were collected using a self-report questionnaire, which may have introduced social desirability bias or common method variance that affected the validity of the findings regarding perceived SDM behavior (Chen et al., 2022). Also, the Observer OPTION^5^ was used to assess self-reported confidence in SDM behavior instead of observing SDM behavior directly. This approach may limit the interpretability and comparability of the findings due to the potential for measurement bias or construct drift. To mitigate this concern, the online survey was administered anonymously to encourage candid feedback and reduce potential pressure from supervisors. In future studies, objective or behavioral indicators may be applied to strengthen the robustness and external validity of the findings. Second, the relatively small sample recruited through convenience sampling from 11 familiar hospitals may have introduced selection bias, which would impact the generalizability of the findings despite the nationwide (all four regions) coverage of the sample. Also, neither the number of eligible nurses in each hospital nor the response rate was recorded and analyzed. Nevertheless, the sample size met the commonly accepted “rule of thumb” that recommends a minimum of 10 participants per predictor variable to ensure sufficient statistical power for the conducted analyses. Furthermore, TPB is a framework grounded in individual beliefs, attitudes, and perceived control regarding a specific behavioral intention and subsequent relevant actions. Also, a number of key organizational-level and socioenvironmental factors (e.g., routine care burden, workload demands, organizational culture and hierarchy, and infrastructural resources) known to affect intended and actual SDM behavior in nurses were unable to be evaluated. Moreover, the complexity involved in both SDM intention and actual engagement among nurses precluded the analyses in this study from being sufficiently comprehensive, despite efforts to account for potential confounding factors (e.g., years of experience practicing SDM, and history of formal SDM training) within the framework (*R* ^2^ = .461 and .514, respectively, in the final regression models). Third, SDM behavior was assessed solely using self-reported measures, and observer-rated assessments were not used. This may limit the interpretability and comparability of the findings due to potential measurement bias and construct drift. Although self-reported and performance-based measures may yield consistent results (Ford et al., 2006), some prior studies on this subject have reported no differences between self-reported and performance-based behavior measures, while others have (Kiechle et al., 2015). Therefore, objective or behavioral indicators may be applied in future studies to strengthen robustness and external validity. Lastly, the cross-sectional design used in this study limited the ability to draw causal inferences between TPB-related factors and SDM behavior. Although direct and indirect paths were examined within the TPB framework using hierarchical linear regression, further path analysis or structural equation modeling will be necessary to elucidate the relationships among TPB-related factors.

### Clinical Implementation

Guided by the findings, strategies designed to enhance attitude toward SDM in nurses should focus on raising awareness and addressing misperceptions. Health care organizations should foster a culture that encourages SDM by enacting supportive policies, demonstrating appropriate leadership, and providing adequate resources. Educational initiatives play a crucial role in shaping the attitude of nurses toward SDM because these initiatives provide essential competencies and encourage reflective practice. In addition, educational interventions should be designed to help nurses perceive SDM as an enhancement to, rather than a burden on, nursing practice. By illustrating how SDM improves communication and trust, which are key components of the nurse–patient relationship, educational programs can help shift nurses’ perspectives and ultimately foster a more positive attitude toward the integration of SDM (Truglio-Londrigan & Slyer, 2018).

Strategies designed to enhance perceived behavioral control should focus on skill development and addressing barriers. Formal education and training programs on SDM can directly improve perceived behavioral control by building related nursing skills and confidence. The findings of a prior study indicate that SDM training programs empower team members to apply SDM more effectively in clinical practice (Alexander et al., 2024). Furthermore, a previous interventional study demonstrated that an interprofessional SDM training program not only enhanced SDM competencies but also enhanced attitude, knowledge, skills, and teamwork in a sample of nurses (Hsiao et al., 2022). An observational study conducted by Milnes et al. (2023) in an intensive care unit revealed that communication training improved significantly the documentation of SDM in patient charts. Another critical strategy for enhancing perceived behavioral control is adopting and using patient decision aids (PDAs) to help structure related discussions and make the SDM process more feasible in practice. In a prior study on SDM implementation, clinicians reported that using PDAs enabled easier and more systematic conversations. Ensuring nurses have easy access to up-to-date PDAs can increase their sense of control over facilitating SDM (Ankolekar et al., 2021). Moreover, organizational barriers such as time constraints, workload pressure, and a hierarchical decision-making structure must be mitigated to further increase the perceived control of nurses over sustainable SDM practice (Bakker et al., 2025; Kienlin et al., 2025)

Although the results of this study indicate subjective norms are not as influential as other TPB-related factors in SDM practice, Légaré et al. (2013) proposed that subjective norms may play a crucial role in promoting SDM by fostering a supportive organizational environment and emphasizing the importance of support from key stakeholders (e.g., employers, colleagues, and patients). When institutional leaders and health care managers visibly prioritize SDM, they send a strong signal that SDM is the expected standard of care (Raleigh et al., 2022). An implementation review reported that, when leaders actively engage in SDM themselves, nursing staff feel supported and are more likely to adopt similar practices (Waddell et al., 2021). Moreover, the presence of respected peers or advocates who actively promote SDM and guide others in its implementation may effectively enhance SDM practice (Raleigh et al., 2022).

As intention to engage in SDM is a strong predictor of perceived SDM behavior, related strategies should focus on enhancing the willingness and motivation of nurses to engage in SDM. Interventions aimed at strengthening behavioral intention should prioritize incentives and social influence. Coulter (2017) identified ethical, financial, and professional incentives as key factors in encouraging clinicians to incorporate SDM into their routine practice. Another strategy is to leverage peer influence and collaborative learning. Peer-led SDM training and communities of practice enable nurses to learn and apply SDM together, share experiences, and support one another, fostering a shared commitment to using these skills (Diouf et al., 2022).

### Conclusions

A TPB framework was employed in this study to explore the potential factors of influence on SDM-related intention and behavior among nurses. The findings suggest that attitude and perceived behavioral control are important predictors of nurses’ intention to engage in SDM, with both intention and perceived behavioral control shown to influence SDM behavior directly. However, subjective norms were found to have relatively less predictive power. Thus, engagement in SDM by nurses may be more closely associated with intrinsic motivation and self-efficacy than with external expectations. Therefore, to more effectively enhance the participation by nurses in SDM, health care institutions should consider focusing on improving attitudes toward SDM, strengthening perceived behavioral control, and fostering behavioral intention.
